# Bayesian Depth-Wise Convolutional Neural Network Design for Brain Tumor MRI Classification

**DOI:** 10.3390/diagnostics12071657

**Published:** 2022-07-07

**Authors:** Favour Ekong, Yongbin Yu, Rutherford Agbeshi Patamia, Xiao Feng, Qian Tang, Pinaki Mazumder, Jingye Cai

**Affiliations:** 1 School of Information and Software Engineering, University of Electronic Science and Technology of China, Chengdu 611731, China; favourekong127@yahoo.com (F.E.); snazzy26@yahoo.com (R.A.P.); fengxiaocd@gmail.com (X.F.); tqtqtang@gmail.com (Q.T.); jycai@uestc.edu.cn (J.C.); 2 Department of Electrical Engineering and Computer Science, University of Michigan, Ann Arbor, MI 48109, USA; pinakimazum@gmail.com

**Keywords:** magnetic resonance imaging (MRI), depth-wise separable convolution, deep learning, Bayesian algorithm

## Abstract

In recent years, deep learning has been applied to many medical imaging fields, including medical image processing, bioinformatics, medical image classification, segmentation, and prediction tasks. Computer-aided detection systems have been widely adopted in brain tumor classification, prediction, detection, diagnosis, and segmentation tasks. This work proposes a novel model that combines the Bayesian algorithm with depth-wise separable convolutions for accurate classification and predictions of brain tumors. We combine Bayesian modeling learning and Convolutional Neural Network learning methods for accurate prediction results to provide the radiologists the means to classify the Magnetic Resonance Imaging (MRI) images rapidly. After thorough experimental analysis, our proposed model outperforms other state-of-the-art models in terms of validation accuracy, training accuracy, *F*1-*score*, recall, and precision. Our model obtained high performances of 99.03% training accuracy and 94.32% validation accuracy, *F*1-*score*, precision, and recall values of 0.94, 0.95, and 0.94, respectively. To the best of our knowledge, the proposed work is the first neural network model that combines the hybrid effect of depth-wise separable convolutions with the Bayesian algorithm using encoders.

## 1. Introduction

The era of deep learning has brought tremendous solutions to challenging tasks that have been previously considered almost impossible or too demanding to deal with in real life. Several methods have been developed and proposed for medical image classification. With the prevalence of deep learning, high-level feature representation of medical images has become more robust, and many state-of-the-art results have been obtained. Magnetic resonance imaging (MRI) is a non-invasive medical imaging technique used to observe various diseases in the body. MRI is an advanced technique with a better resolution with soft tissues than other forms of medical imaging. Radiologists and health experts use magnetic resonance examination to diagnose diseases, detect abnormal tissues or tumors, and guide surgical procedures. A brain tumor is caused by forming a significant mass of abnormal cells inside or around the brain. These cells must be detected promptly because the cancer is fatal if not analyzed and treated, affecting the brain processing functions and the patient’s holistic health. MRI is the most common technique for obtaining and diagnosing a specific type of brain tumor.

According to the World Health Organization (WHO), brain tumors can be classified into various types based on their cell origin. These types include meningioma, pituitary adenoma, brain stem gliomas, oligodendrogliomas, astrocytoma, ependymomas, glioblastoma, etc. The meningioma type of brain tumor is often benign and can be treated if detected early. They are low-grade tumors, which can be non-cancerous cells and are very unlikely to spread. Meningioma tumor is slowly formed in the membrane surrounding the spinal cord and the brain. The pituitary adenoma [1] type of brain tumor grows slowly and is usually harmless in some cases. The tumors develop from tissues in the pituitary gland, located at the base of the brain. This tumor type is prevalent as the pituitary gland is responsible for controlling other glands in the body. The glioma [2] type of tumor is developed from the glial cell and can be seen from a biopsy. Brain stem gliomas are found in the lower part of the brain connected to the spinal cord, which can be challenging to treat as they affect the basic functionalities of the brain and central nervous system.

A Bayesian Neural Network (BNN) is an artificial neural network with priors, built using stochastic processes and trained using Bayesian Inference. A particular type of Artificial Neural Network (ANN) is a Stochastic Neural Network. Stochastic neural networks are built by feeding stochastic components into the neural network using stochastic weights to simulate a set of models *θ* with their respective probability distribution p(θ). Stochastic neural networks are used to estimate the uncertainty in the model prediction. In other words, the predictions for each model are aggregated after the models are trained to understand the predictions’ uncertainty better. Bayesian estimation of unknown parameters is essential for estimating the uncertainty and confidence in these output decisions. The prior distributions are first introduced over the network weights. Then the posterior distributions are estimated subsequently.

Bayesian Neural Networks are made up of neural networks with a prior distribution on their weights [3], as well as a probabilistic (or statistical) model, which forms the core of this integration. This combination helps explore the strength and capabilities of both neural networks and probabilistic modeling. Bayesian networks can approximate functions using probabilistic models that allow direct specification. Valuable data can be generated from specific parameterization in statistical modeling. In the prediction stage, the statistical models ensure probabilistic guarantees on the model and generate a posterior distribution of the parameters learned from previous examples’ observations. The nature and distribution of the previously learned parameters can be deduced in the parameter space. Bayes Theorem helps support the development of top machine learning algorithms and provides a critical framework to analyze stochastic neural networks and train network models. We can perform model comparison and selection using this approach without a separate cross-validation dataset. Regularization is also an excellent technique to reduce errors in the output distribution of the training data.

A deep neural network is an artificial network model with an input unit, multiple hidden layers, and an output layer. Complex data models can be created with many hidden layers and can perform better than a similar shallow network model [4]. Convolutional Neural Network (CNN) models consistently outperform other classical machine learning techniques (including support vector machines, random forest, and K-nearest neighbors) since 2012 when AlexNet won the ImageNet Large Scale Visual Recognition Competition [5]. However, while CNN has achieved state-of-the-art performance in medical image segmentation, image recognition, and medical image classification, there are few works addressing image registration using CNN.

For medical image classification, a recent study [6] detailed the image-based brain tumor segmentation using machine learning classifiers. Another study [7] proposed a 3D CNN model for breast cancer detection using Clinical Image data.

Many works have been done on medical imaging, magnetic resonance imaging (MRI) using reinforcement learning, and other deep learning techniques. The major challenge is the data acquisition process, which is very time-consuming and could be tiresome for hospital patients. According to a study on the application of compressed sensing for rapid MRI [8], the average patient waiting for the full MRI scan takes about 15 min to 60 min while remaining still in a given position, which is because the data is mainly sampled sequentially in K-space or frequency domain. To this end, a practical solution to this tedious process is Compressed-Sensing MRI (CS-MRI) to accelerate data acquisition and reduce the time and hardware resources used.

In recent years, reinforcement learning has been applied to the medical field in MRI reconstruction, medical image processing, and so on. In a recent study, a detection agent was trained using Deep Reinforcement Learning (DRL) for localizing landmarks in 3D CT images [9]. DRL swiftly combines Reinforcement Learning with Deep Neural Networks, making it applicable to more complex problems (including game theories and human-level controls) as well as producing high-level performances [10]. However, deep reinforcement learning has many drawbacks, including the need for lots of data to learn, lots of computation with high computational costs, sample inefficiency, and the tendency to rationalize the information provided.

Regarding machine learning, many works have been proposed for the diagnosis of various medical conditions, including learning techniques for the diagnosis of Alzheimer’s disease [11], classification of breast tumors [12], predicting outcomes of clinical trials of prostate cancer [13], as well as many other related works.

The previous work on multispectral tissue classification [14], using statistical pattern recognition techniques, represented one of the most seminal works leading up to today’s machine learning in medical imaging segmentation. There are different contributions regarding supervised and unsupervised machine learning approaches for magnetic resonance image segmentation, and classification tasks [15]. A range of segmentation and classification methods, such as deep CNN models, have been proposed for brain image analysis on MRI [16,17].

Many algorithms, including Principal Component Analysis (PCA), Discrete Wavelength transform (DWT) [18], Support Vector Machines (SVM), etc., have been used in different works for medical imaging. Table 1 shows different related works on Reinforcement Learning, CNN research trends, medical image classification, prediction, and segmentation tasks, as well as their contributions.

Another challenge of using deep reinforcement learning is that the time taken during the training process is too much compared to other deep learning forms.

The main difference between our work and other deep CNN reinforcement learning models is that our model is the first neural network model that combines the hybrid effect of depth-wise separable convolutions with the Bayesian algorithm using encoders. Other conventional CNN architectures deploy point-wise convolutions with a higher computational cost, but our work uses depth-wise separable convolutions with the Bayesian algorithm to ensure accurate predictions.

The existing problems from previous reinforcement learning models and deep neural network models include training instability, interference and exploration problems, sample inefficiency, safety constraints, real-time inference problems, and delayed reward functions. On the one hand, we present a new Bayesian deep convolutional network that can solve the disadvantages of previous CNN and reinforcement learning models and combine and use the prediction advantages of the existing models. On the other hand, we compare our results with existing works regarding classification, prediction metrics, and accuracy. These are the motivations to introduce the related works in medical imaging and other CNN trends.

## 2. Materials and Methods

### 2.1. Problem Statement and Contributions

Although there are significant advances in deep learning, reinforcement learning, and machine learning fields, the application in the medical field is still very limited due to the few datasets and the private nature of this field. However, in this work, we apply the advantages of the Bayesian neural network, which requires a small number of probability variables to predict the continuous output confidently. The Bayesian model can also solve queries in the joint distribution by adding all relevant entries. Since Bayesian classifiers and networks are still traditional machine learning methods, they can complement the depth-wise separable convolutions in deep CNN models. They can be used to predict and classify medical images accurately.

Although medical image processing is very time-consuming, different techniques used to increase the speed of the process have been employed. Machine learning and artificial intelligence have rapidly advanced in different fields, especially medical imaging. Different techniques of artificial intelligence and machine learning have played essential roles in the medical field, including medical image processing, image segmentation [16], image interpretation and interpolation, computer-aided diagnosis, image fusion, image classification, and so on. Several methods have been proposed to improve MRI image detection and classification accuracy.

In recent years, many deep learning approaches have been proposed for medical image classification and segmentation. With the prevalence of deep learning, high-level feature representation of medical images has become more robust, and many state-of-the-art results have been obtained. The innovation of this work is the proposed model, which can accurately perform classification with a high degree of probabilistic prediction accuracy. The main contributions to this work include:

1. A new method for proper medical image classification and prediction is proposed using depth-wise separable convolutions instead of the conventional standard point-wise convolutional layers;
2. By combining the Bayesian algorithm to depth-wise separable convolutions, the proposed method obtains better results in terms of different evaluation metrics;
3. The proposed model mainly focuses on improving the accuracy of MRI image detection and ensuring efficient classification promptly to aid radiologists in obtaining an accurate model for medical diagnosis. This model ensures efficient predictions for the tumor type by implementing medical image registration and bias field correction on the datasets.

### 2.2. Datasets

The dataset for this work is obtained from the repository of the BRAIN Initiative Neuroscience Information framework [29], and the Multimodal Brain Tumor Image Segmentation Benchmark (BRATS) 2015 dataset [30].

The BRATS dataset is from the competition on brain tumor segmentation. This dataset consists of patients with glioblastoma and lower grade glioma, but we only acquired the part for glioma in this research work.

The healthy slices of MRI images were obtained from the IXI dataset, containing T1 and T2 weighted images from healthy and normal subjects [31]. The images obtained were in NIFTI format, so they had to be converted into PNG format for recognition by our model.

The Glioma class is obtained from the BRATS 2015 dataset consisting of high-grade and low-grade gliomas, while pituitary and meningioma classes are obtained from the image database [32] containing contrast-enhanced MRI images.

The dataset has four main classes: no tumor, pituitary tumor, meningioma tumor, and glioma tumor. This is why the categorical class mode is used for multi-class classification. The overall dataset consists of 4000 images, with each class having 1000 images. The training and testing sets were split in the ratio of 4:1, with 80% of the data used for training and 20% of the data used for testing. However, for models that require a training set, test set, and validation set, the data is divided into 80% for the training set, 10% for the test set, and 10% for the validation set. There are 3200 training samples and 800 test samples. The preferred optimizer used in this work is the Adam optimizer because it is easy to implement and has a lower computational cost than other optimizers. In addition, due to the noise in some of the data, the Adam optimizer proved to be the best when filtering out the noise, and it works well with large datasets. This optimizer requires little memory for computation and deals with sparse gradients problems. Adam optimizer is used in the training process, with a learning rate of 0.0001. This work uses the rectified Linear unit activation function and SoftMax activation for the final output classes.

### 2.3. Mathematical Definitions and Algorithms

Let *M* and *N* represent the number of input channels and output channels of a convolutional layer with Kernel *K*, the input of the feature map *F* can be represented as $I_{F}\times I_{F}\times M$, where *I_F_* represents the spatial width and spatial height of *F*. The size of *K* is calculated as $I_{K}\times I_{K}\times M\times N$, where *I_K_* is the spatial dimension of the kernel, which must be square. For standard point-wise convolutions, the computational cost can be computed as $I_{K}\cdot I_{K}\cdot M\cdot N\cdot I_{F}\cdot I_{F}$.

Let (*i*, *j*) represent the spatial coordinate in the receptive field of size *k*, *y* represent the output label space, *m* represent the number of filters applied in Kernel *K*, and *W* represent the convolutional weight applied, then the point-wise convolutional process **P** [33] is given by:

(1) $$P{\left(W,y\right)}_{\left(i,j\right)}=\sum_{m}^{M}W_{m}\cdot y_{\left(i,j,m\right)}$$

Assuming the network model has *L* depth-wise convolutional layers, the depth-wise convolutional process **D** in layer *l* performs element wise multiplication (denoted by ⊙), as shown below:

(2) $$D{\left(W,y\right)}_{\left(i,j\right)}=\sum_{k,l}^{K,L}W_{k,l}⊙y_{\left(i+k,j+l\right)}$$

However, in our model, we apply depth-wise separable convolutions to restrict the relationship between the output channels and the kernel. The point-wise and depth-wise convolutions all make up the term depth-wise separable convolutions. Point-wise convolutions are just normal 1 × 1 convolutions with the stride of 1. For depth-wise convolutions, there is no dependency on the number of output channels so that the computational cost can be computed as $I_{K}\cdot I_{K}\cdot M\cdot I_{F}\cdot I_{F}$. Depth-wise separable convolutions have lower computational costs, about nine times less than standard convolutions. The depth-wise separable convolutional process **Z**, with spatial coordinate (*i*, *j*), point-wise convolutional weight $W_{p}$, and depth-wise convolutional weight $W_{d}$, is given by:

(3) $$Z{\left(W_{p},W_{d},y\right)}_{\left(i,j\right)}=P\left(i,j\right)\left(W_{p},D\left(i,j\right)\left(W_{d},y\right)\right)$$

The training algorithm for the Bayesian model with deep priors is given in Algorithm 1. The input parameters are the MRI dataset to be trained, which is further split into the test and training sets. Encoders are used, and the Evidence Lower Bound (ELBO) function is used for computation. The main reason for employing Bayesian modeling is to ensure that the prior beliefs influence the posterior beliefs. Let Pr(w) denote the weights of the prior distribution, α denote the first hyperparameter of a prior distribution over another parameter, $E_{w}$ denote the estimator of the weights such that the prior becomes a Gaussian function, and let $Z_{w}$ denote the normalizer, then Pr(w) can be represented as:

(4) $$Pr\left(w\right)=\frac{e^{-\alpha E_{w}}}{Z_{w}\left(\alpha \right)}$$

(5) $$where\;Z_{w}\left(\alpha \right)=\int \left(e^{-\alpha E_{w}}\right)dw$$

**Algorithm 1:** Bayesian Training Algorithm with Depth-wise Separable Convolutions
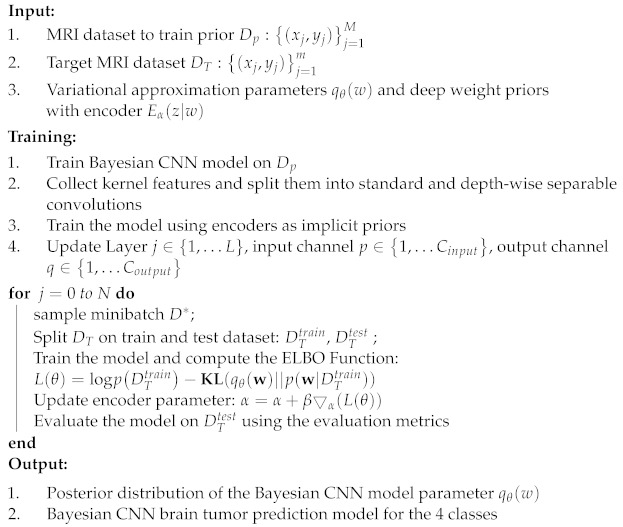


### 2.4. Training Algorithm

Due to the intractable nature and lack of analytical solutions of the posterior inference methods, the need for variational inference and other practical approximation techniques arises. The idea behind variational inference is translating the inference of the posterior directly into an optimization problem, which could either be a minimization or maximization problem. Modern Deep neural network architectures have multiple layers of linear and nonlinear transformations; thus, Variational inference can be used to scale large approximations of these architectures. The emerging challenge with this approach is that the propagation of probability distributions is introduced over the weights through several deep layers. Bayesian Convolutional Neural Network (BCNN) is used for various deep learning tasks such as image Super-Resolution [34], Generative Adversarial Network modeling [35], and various image classification tasks. The Kullback–Leibler (KL) divergence parameter [36] between the true posterior p(w|D) and the variational approximation $q_{\theta }\left(w\right)$ or variational distribution q(w|θ) can be used to measure the closeness of the approximations with respect to *θ*, where the training dataset is represented as:

(6) $$D=\left\{x^{\left(i\right)},y^{\left(i\right)}\right\}$$

The definition of KL divergence is given by:

(7) $$KL\left(q\left(x\right)||p\left(x\right)\right)=-\int q\left(x\right)log\left(\frac{p(x)}{q(x)}\right)$$

The cost function or variational free energy is expressed as:

(8) $$F\left(D,\theta \right)=KL(q_{\theta }\left||p\left(w\right)\right)-E_{q_{\theta }\left(w\right)}\log\left(p\left(D|w\right)\right)$$

### 2.5. Architecture of the Proposed Model

**Figure 1:**
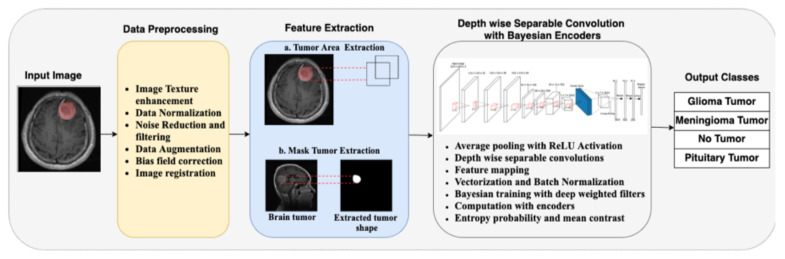
Architecture of the proposed model.

The architecture of the proposed model in this work is illustrated in Figure 1. We take the input MRI dataset for the proposed model and perform specific preprocessing tasks on the original images. The images are rescaled to the desired input format, and data normalization is performed. Since there are noises in the data, we performed data cleaning to reduce and filter out the unwanted noise in the data. Next, data augmentation is performed on the data, including horizontal and vertical shifts, rotations, image brightening, image enlargement, horizontal flips, and vertical flips. Bias field correction is also performed using the fuzzy C-means clustering algorithm. The final preprocessing step is image registration with reference images and other images that should be aligned correspondingly.

After preprocessing, specific feature extraction is performed on the image. This part is divided into tumor area extraction and mask tumor extraction with ground truth. The extracted features are used to train the neural network. The training phase uses depth-wise separable convolution and Bayesian training with encoders. Average pooling is used in this work instead of the maximum pooling used in conventional CNN architectures. The depth-wise separable convolutions involve the aggregation of point-wise convolutions and depth-wise convolutions. Vectorization is performed as the next step, along with feature mapping of the desired tumor region. Bayesian training is done with Algorithm 1, using the model’s deep-weighted filters and weighted priors. Encoders are also used in this step, and the entropy probability and mean contrasts are computed automatically by the model. For validation, an input image is selected randomly from the test set. Then, the model predicts the class to which the image belongs. If the image is not a brain tumor MRI image, it will automatically refer to the output class of “No tumor”.

**Figure 2:**
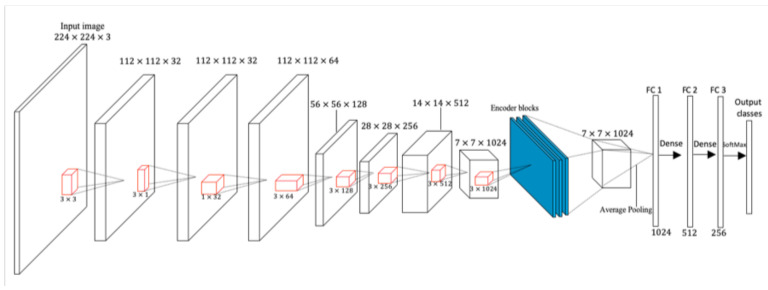
Layered representation of the depth-wise separable features.

In Figure 2, the whole convolution process using depth-wise separable convolution with Bayesian encoders is illustrated. The ReLU activation function is used after every point-wise convolutional layer, while the Softmax classifier is used in the output layer. The red cuboids after every depth-wise separable convolution in Figure 2 represent the kernels.

The input layer of this model takes the shape of 224 × 224 × 3, with 3-dimensional color channels. The batch sizes of 32 and 16 are both used due to the capacity of the graphics processing unit. Larger batch sizes were not used in this work because of the high tendency to obtain redundant results. The number of iterations is set to 100 epochs and trained for 671 seconds. Each epoch represents the complete cycle on the whole dataset. Dropouts were used in the fully-connected layers, with a maximum dropout probability of 50%. Table 2 lists the proposed work’s input and training parameters.

## 3. Results

### 3.1. Experimental Setup

In this work, several implementation tools are used, such as Keras, TensorFlow, PyTorch, and Caffe Application Programming Interface. The main programming language used is Python. The proposed model is trained on the brain tumor MRI dataset.

The codes used in this work were implemented using the GeForce RTX Graphical Processing Unit (GPU) due to the high performance and speed of training. The processor used is an Intel Core i-7 central processing unit with an operating system memory of about 1.2 TB. The Google Colaboratory [37] tool is also used for running the codes because of the speed of its processor and in-built libraries. The CUDA driver is used for the training, supported by NVIDIA for computer graphics compilation. The CUDA version used is 9.0.176, and it helped to ensure maximum utilization of the GPU resources. PyTorch 1.2 is used with Python 3.9.1 for training. Furthermore, the model is trained with the Keras framework using the TensorFlow backend.

### 3.2. Evaluation Criteria

Several evaluation metrics are used in this work, including accuracy, *F*1-*score*, recall, and precision. This study also uses confusion matrices to evaluate the performance algorithms. The confusion matrix is an error matrix that allows visualization of the model’s performance. In the equations below, *TP*, *TN*, *FP*, and *FN* represent True Positive, True Negative, False Positive, and False Negative, respectively.

(9) $$Accuracy=\frac{TP+TN}{TP+TN+FP+FN}$$

(10) $$Precision=\frac{TP}{TP+FP}$$

(11) $$Recall=\frac{TP}{TP+FN}$$

(12) $$F1-score=\frac{2*Precision*Recall}{Precision+Recall}$$

For *TP*, the model predicts a positive result in the presence of the tumor, such that tumor samples are correctly predicted as tumor samples. For *TN*, the model predicts a negative result in the absence of the tumor, such that non-tumor samples are correctly predicted as non-tumor samples. For *FP*, the model predicts a positive result without the tumor, such that non-tumor samples are falsely predicted as tumor samples. For *FN*, the model predicts a negative result in the presence of the tumor, such that tumor samples are falsely predicted as non-tumor samples.

Entropy is the measure of randomness or uncertainty in predicting the data. The entropy value ranging from [0–1] reflects the level of uncertainty disorder, depending on the number of classes present in the dataset. The main goal of this neural network model is to reduce the uncertainty of the order and keep the entropy as low as possible. Let $p_{i}$ represent the frequentist probability of an element *i*, then the equation for calculating the data entropy is given below.

(13) $$H=\sum_{i=1}^{c}-p_{i}\log_{2}p_{i}$$

### 3.3. Accuracy and Loss Representations

**Figure 3:**
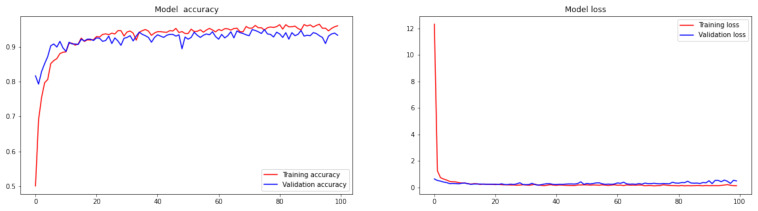
Accuracy and loss representation for the proposed model.

**Figure 4:**
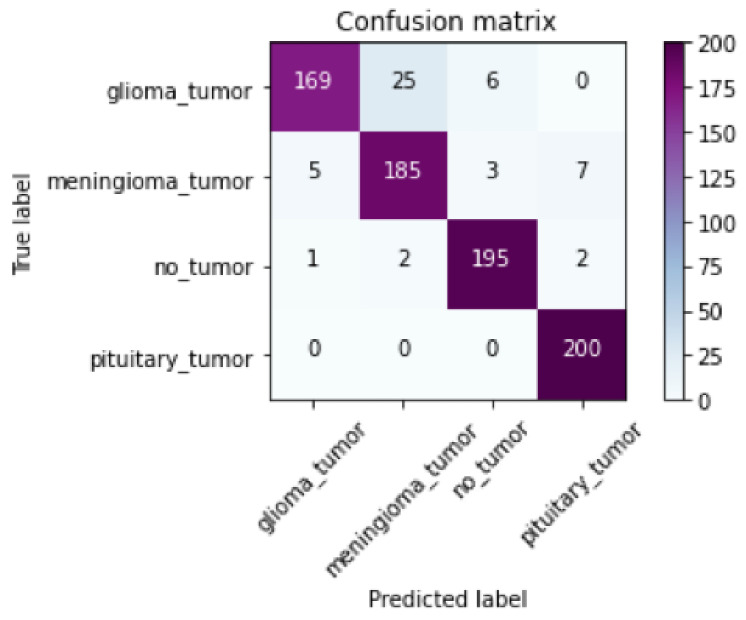
Confusion matrix for the proposed model.

After training our proposed model with the Bayesian training algorithm and depth-wise separable convolutional layers, we obtained the graphs for training and validation for the proposed model, along with the confusion matrix, as shown in Figure 3 and Figure 4. Our model’s training accuracy and loss are 99.28% and 0.0269, respectively. Our model’s validation accuracy and loss are 94.38% and 0.4082, respectively.

Our model also correctly classified all the images in the pituitary tumor class, as there was no misclassification in this class, as can be seen from the confusion matrix in Figure 4. Our model obtained a weighted average of 0.99 for precision, recall, and *F*1-*score*. However, for validation, our model obtained a weighted average precision value of 0.95, an average recall value of 0.94, and an average *F*1-*score* of 0.94.

### 3.4. Bayesian Prediction Results with Different MRI Images

**Figure 5:**
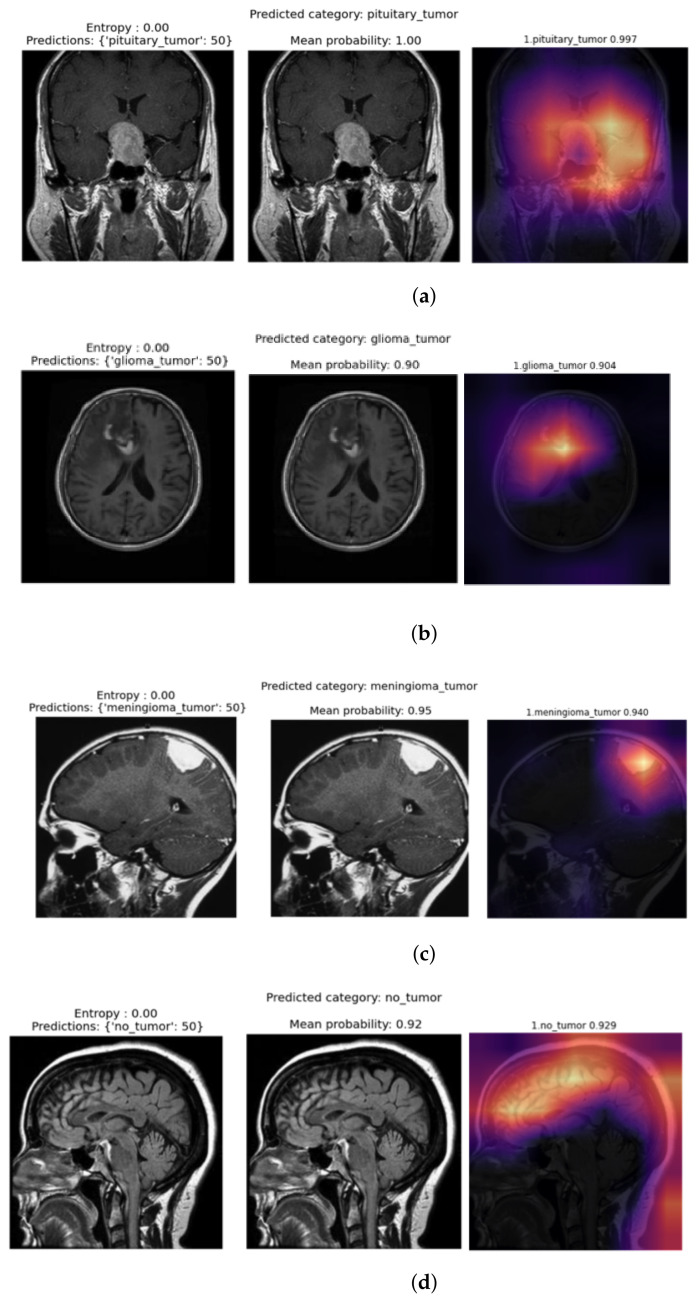
Prediction categories for the four classes in the dataset. (**a**) Pituitary tumor: prediction (left), mean probability (middle), and heat map (right). (**b**) Glioma tumor: prediction (left), mean probability (middle), and heat map (right). (**c**) Meningioma tumor: prediction (left), mean probability (middle), and heat map (right). (**d**) No tumor: prediction (left), mean probability (middle), and heat map (right).

**Figure 6:**
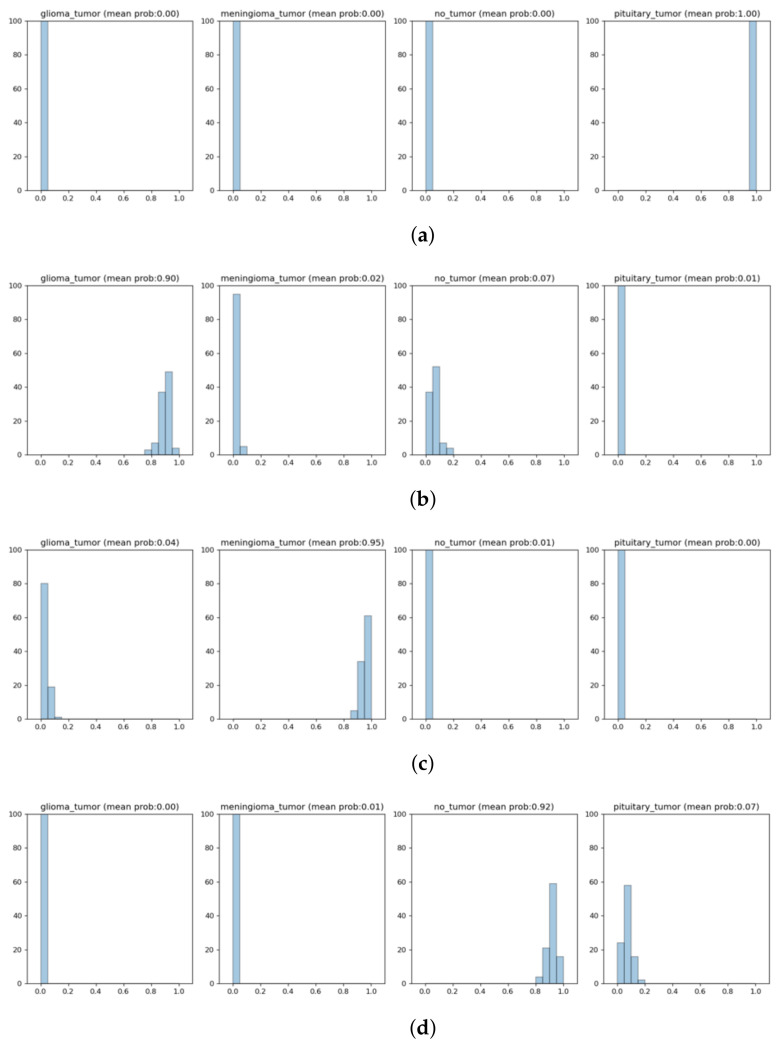
Mean probabilities for the four classes in the dataset. (**a**) Mean probability graphs: “pituitary tumor” = 1.00. (**b**) Mean probability graphs: “glioma tumor” = 0.90. (**c**) Mean probability graphs: “meningioma tumor” = 0.95. (**d**) Mean probability graphs: “no tumor” = 0.92.

In this part, we describe the prediction categories for the four classes in the dataset, as shown in Figure 5a–d. Additionally, we describe the mean probabilities for the four classes in the dataset, as shown in Figure 6a–d.

The model predicts the category the input image belongs to, then calculates the entropy value and the Bayesian mean probability that the prediction is correct. The tumor heat map area for the pituitary tumor is also plotted on the far right of Figure 5a.

The confusion matrix for our model explains that few glioma tumor images are incorrectly predicted as “meningioma tumor” and “no tumor” classes. This is due to the uncertainty and resemblance of these medical images. However, our model predicts the input image correctly to be a glioma tumor, with a mean probability of 0.90. Figure 5b gives the category, entropy, mean probability, and heatmap for Glioma tumor.

Additionally, some meningioma tumor images are incorrectly predicted as “glioma tumor” and “no tumor” classes. However, our model predicts the input image correctly to be a glioma tumor, with a mean probability of 0.95. Figure 5c gives the category, entropy, mean probability, and heatmap for the meningioma tumor region. The confusion matrix shows that some healthy MRI images are incorrectly categorized as tumor classes. However, our model predicts the input image correctly to be “no tumor”, with a mean probability of 0.92. Figure 5d gives the category, entropy, mean probability, and heatmap for the “no tumor” region. It is worth noting that if the entropy is low, then the model’s uncertainty will also be low. If the mean probability is 1.0, then the model is confident that the prediction is accurate and belongs to the class category predicted.

The differences between Figure 5a–d lie in the heatmap of the tumor area (located at the right of each figure), the mean probabilities provided in the middle, and the prediction category of the model (provided at the left of each figure). The mean probabilities for all four classes (in the middle of Figure 5a–d) are further obtained and plotted separately in Figure 6a–d, respectively.

In Figure 6a, the mean probability is 1.0 for “pituitary tumor”, while it remains 0.0 for the other three classes. This is a perfect prediction. In Figure 6b, the mean probabilities are plotted for all four classes. The value is 0.9 for “glioma tumor”, 0.02 for “meningioma tumor”, 0.07 for “no tumor”, and 0.01 for “pituitary tumor”.

In Figure 6c, the mean probabilities are plotted for all four classes, and the value is 0.95 for the “meningioma tumor” class, 0.04 for the “glioma tumor” class, 0.01 for the “no tumor” class, and 0.00 for the “pituitary tumor” class. In Figure 6d, the mean probabilities are plotted for all four classes, and the value is 0.92 for the “no tumor” class, 0.07 for the “pituitary tumor” class, 0.01 for the “meningioma tumor” class, and 0.00 for the “glioma tumor” class.

### 3.5. Comparison of All Models

In this section, we compare all the models to our proposed model. We plot the bar chart representation and give the tabular comparisons of the models and their recall values, precision scores, *F*1-*scores*, accuracy, and loss values. Table 3 gives the tabular comparison of all models used in this work.

In AlexNet training, we converted our input images to 227 × 227, trained with rectified Linear Unit activation, and added dense layers with neurons 4096, 1000, and an output of 4 classes. We trained with Adam optimizer and performed preprocessing on the dataset, including reshaping, rotation, horizontal and vertical flip, and zooming with a specific range.

In VGG16 training, we converted our input images to 224 × 224, trained with rectified Linear Unit activation. There are 13 convolutional layers, 3 fully-connected layers, with neurons 4096, 4096, and an output of 4 classes. We trained with Adam optimizer and performed preprocessing on the dataset.

In MobileNet training, we used the input dimension of 224 × 224 for the images. The activation function used was Rectified Linear Unit. There are 28 layers in the MobileNet architecture, with an average pooling layer, 1024 neurons in the fully-connected layer, and an output of 4 classes.

The ResNet model consists of 5 stages, each having different convolutional and identity blocks. Each identity block in the model has three convolutional layers, while the convolutional block also has three convolutional layers. Batch normalization is used after every layer, and the ReLU activation function is used. There are about 47 million trainable parameters in this model.

In the Standard CNN model with Point-wise Convolutional layers (PW-CNN), there are six point-wise convolutional layers, one dense layer, and four classes output of four classes. Batch normalization is used with the ReLU activation function. Adam optimizer is also used to train the model over many iterations.

In the previous work using Kernel Extreme Learning Machines (KELM) [38], the 10-fold cross-validation method is used for testing, and the dataset is divided into 80% for the training set and 20% for the validation set.

In the previous work using capsule network for brain tumor classification [39], the k-fold cross-validation method is not used for testing, and the dataset is also divided into 80% for the training set and 20% for the validation set.

**Figure 7:**
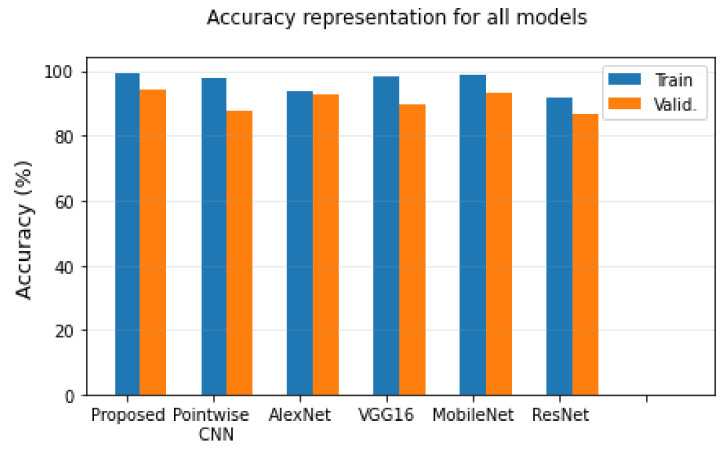
Accuracy representations for all models.

From Table 3, the best values from the experimental criteria are given in bold. Our model outperforms other models in terms of training accuracy (Tr. Acc), validation accuracy (Val. Acc), *F*1-*scores*, and precision. Our model obtained the joint-best recall score of 0.94 and the MobileNet model. The comparison of the training and validation accuracy for all the models is given in Figure 7.

### 3.6. Ablation Study

**Figure 8:**
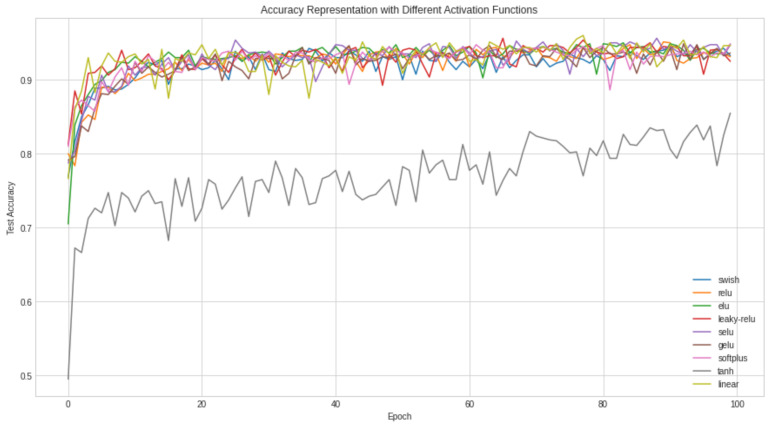
Accuracy representations of the proposed model with different activation functions.

We tested our model’s efficiency using various activation functions such as Rectified Linear Unit (ReLU), Leaky Rectified Linear Unit (LReLU), Swish activation function, Exponential Linear Unit (ELU), Scaled Exponential Linear Unit (SELU), Gaussian Error Linear Unit (GELU), Hyperbolic Tangent, Linear activation, and Softplus activation. The graphical representation of the proposed model trained with different activation functions is given in Figure 8. The SeLU activation has the advantage of internal normalization. The TanH activation has the vanishing gradient problem, which is not the case in ReLU activation. GELU also avoids the vanishing gradient problem, although it is not commonly used in practice. For the Softplus activation, the computation is relatively large and expensive when obtaining the back-propagation gradient. The first derivative of Softplus is the sigmoid function (used for binary classification), which makes it soft saturated. For linear activation, the derivative remains constant, and the error does not improve since the gradient is the same. From Table 4, although the LReLU activation had a higher training accuracy than other activation functions, it is very inconsistent. It produces a very high loss value, which is not applicable for training our brain tumor images. The Leaky ReLU activation function saturates for large negative values, rendering them inactive. In other words, if the input value is negative, then the gradient will be represented as the hyperparameter *α*. ELU activation is computationally expensive because of the exponential term, although it gives a high precision value as the ReLU function. The Swish activation function is unbounded above like the ReLU function and bounded below, although it is computationally expensive. Our proposed model uses the RELU activation to ensure computational effectiveness, reduce time complexity, and provide sparsity in the network model, thereby reducing over-fitting and noise. The non-linear nature of ReLU makes the network model adaptable to learning complex patterns. Table 4 compares the proposed model with different activation functions.

## 4. Discussion

In this work, a fine-grained method for classifying brain tumors using deep learning and the Bayesian method has been proposed. This approach breaks the barrier of simply relying on the conventional neural network classification methods and can also be applied to different medical imaging tasks. Our proposed method combines the Bayesian algorithm using deep filters and depth-wise separable convolutional neural networks with learnable features. We test and compare our deep learning model with benchmark models, including AlexNet, ResNet, MobileNet, VGG16, and Conventional CNN, with point-wise convolutions. Then, we evaluate the models in terms of probabilistic prediction, accuracy, recall value, precision, and *F*1-*score*. Experimental analysis shows that our model outperforms other network models in precision, recall, *F*1-*score*, training accuracy, and validation accuracy. The high performances of 99.03% training accuracy and 94.32% validation accuracy give us high confidence that this work can be used in real-life scenarios and radiology. To the best of our knowledge, the proposed work is the first neural network model that combines the hybrid effect of depth-wise separable convolutions with the Bayesian algorithm using encoders. In future works, we will explore other data augmentation techniques to increase the size of the data and ensure the model’s generalization capability. Another future improvement would be to adopt a three-dimensional system to ensure that this model can be used in clinical trials and adjust to real-world brain tumor classification scenarios. This will help us compare our system model to other existing models in real life, compare the pros and cons of each system, and finally upgrade our proposed network model to complete optimization. We will employ automated techniques using deep learning and artificial intelligence to resist adversarial attacks against our model if used in clinical phases.

## Figures and Tables

**Table 1:** Related works on MRI prediction and classification, CNN trends, medical imaging, and their contributions.

Papers	Features	Main Contributions
[19]	Fully optimized deep CNN for multi-classification of brain tumor MRI	Three fully automatic CNN models for multi-classification of brain tumors using publicly available datasets.
[20]	Hybrid fuzzy brain-storm optimization algorithm for the classification of brain tumor MRI	Fuzzy brain-storm optimization algorithm for medical image segmentation and classification.
[21]	Evaluation and classification of the brain tumor MRI using machine learning technique	Machine-Learning-Technique (MLT) to evaluate and classify the tumor regions in brain MRI slices.
[22]	Ensemble deep features and ML classifiers for MRI-Based brain tumor classification	Brain tumor classification using an ensemble of deep features and machine learning classifiers
[23]	MRI brain tumor classification using CNN	CNN approach to categorize brain MRI scan images into cancerous and non-cancerous
[24]	Transfer learning using CNN architectures for MRI brain tumor classification	Deep transfer learning for feature extraction using deep pre-trained CNN architectures
[25]	MRI brain tumor classification using Deep reinforcement learning	Reinforcement learning for MRI brain tumor image classification
[26]	Capsule Networks (CapsNets)	Brain tumor classification using capsule networks
[27]	CNN + Multi-step Reinforcement Learning (MRL)	CNN-MRL Hybrid model for Image Processing
[28]	Object detection with MobileNet	Driver drowsiness detection app for smartphones based on deep learning features

**Table 2:** Input parameters for training the proposed model.

Parameters	Values
Input shape	224 × 224 × 3
Batch size	32
Number of epochs	100
Number of training samples	3200 images
Number of test samples	800 images
Training time	671 s
Output classes	4
Class mode	Categorical (Multi-class classification)
Optimizer	Adam
Learning rate	0.0001
Activation function	ReLU, SoftMax
Dropout Probability	50%

**Table 3:** Comparison of the proposed model to various models.

Models	Training Acc. (%)	Validation Acc. (%)	Precision	*F*1-*Score*	Recall
ResNet50	91.91	86.58	0.88	0.86	0.87
Alexnet	93.60	92.75	0.94	0.93	0.93
VGG16	98.50	89.51	0.92	0.91	0.91
MobileNet	98.96	93.42	0.94	**0.94**	**0.94**
PW-CNN	97.86	87.87	0.88	0.88	0.88
**Ours + ReLU**	**99.19**	**94.38**	**0.95**	**0.94**	**0.94**
Pashaei [38]	93.68	-	0.94	0.93	0.91
Kurup [39]	92.60	-	0.92	0.93	**0.94**

**Table 4:** Comparison of the proposed model using different activation functions.

Activation Functions	Training Acc. (%)	Validation Acc. (%)	Precision	*F*1-*score*	Recall
Swish	98.87	93.75	0.94	0.93	0.93
SELU	98.22	92.50	0.94	0.92	0.92
ELU	99.72	93.50	**0.95**	0.93	0.92
GELU	99.28	93.00	0.93	0.93	0.93
LReLU	**99.97**	94.00	0.94	**0.94**	**0.94**
TanH	86.81	84.13	0.85	0.84	0.83
Linear	99.19	92.75	0.94	0.93	0.93
Softplus	99.37	92.87	0.94	0.93	0.93
ReLU	99.19	**94.38**	**0.95**	**0.94**	**0.94**

## Data Availability

The dataset for this work is obtained from the repository of the BRAIN Initiative Neuroscience Information framework [29] and the BRATS 2015 dataset [30]. The BRATS dataset is from the competition on brain tumor segmentation. This dataset consists of patients with glioblastoma and lower grade glioma, but we only acquired the part for glioma in this research work. The glioma class in our dataset is obtained from the BRATS 2015 dataset. The pituitary and meningioma classes in our dataset are obtained from the image database [32] containing contrast-enhanced MRI images.

## References

[1] Nishioka H., Inoshita N. (2017). New WHO classification of pituitary adenomas (4th edition): Assessment of pituitary transcription factors and the prognostic histological factors. Brain Tumor Pathol..

[2] Iuchi T., Sugiyama T., Ohira M., Kageyama H., Yokoi S., Sakaida T., Hasegawa Y., Setoguchi T., Itami M. (2018). Clinical significance of the 2016 WHO classification in Japanese patients with gliomas. Brain Tumor Pathol..

[3] Hinton G.E., Neal R. (1995). Bayesian Learning for Neural Networks.

[4] Hinton G.E., Salakhutdinov R. (2006). Reducing the Dimensionality of Data with Neural Networks. Science.

[5] Krizhevsky A., Sutskever I., Hinton G.E. (2012). ImageNet classification with deep convolutional neural networks. Commun. ACM.

[6] Preetika B., Latha M., Senthilmurugan M., Chinnaiyan R. MRI Image based Brain Tumour Segmentation using Machine Learning Classifiers. Proceedings of the 2021 International Conference on Computer Communication and Informatics (ICCCI).

[7] Haq A.U., Li J.P., Saboor A., Khan J., Zhou W., Jiang T., Raji M.F., Wali S. 3DCNN: Three-Layers Deep Convolutional Neural Network Architecture for Breast Cancer Detection using Clinical Image Data. Proceedings of the 2020 17th International Computer Conference on Wavelet Active Media Technology and Information Processing (ICCWAMTIP).

[8] Hamamci A., Kucuk N., Karaman K., Engin K., Ünal G. (2012). Tumor-Cut: Segmentation of Brain Tumors on Contrast Enhanced MR Images for Radiosurgery Applications. IEEE Trans. Med Imaging.

[9] Ghesu F.C., Georgescu B., Mansi T., Neumann D., Hornegger J., Comaniciu D. An Artificial Agent for Anatomical Landmark Detection in Medical Images. Proceedings of the MICCAI.

[10] Mnih V., Kavukcuoglu K., Silver D., Rusu A.A., Veness J., Bellemare M.G., Graves A., Riedmiller M.A., Fidjeland A., Ostrovski G. (2015). Human-level control through deep reinforcement learning. Nature.

[11] Tanveer M., Richhariya B., Khan R.U., Rashida H.S., Khanna P., Prasad M., Lin C.T. (2020). Machine Learning Techniques for the Diagnosis of Alzheimer’s Disease. ACM Trans. Multimed. Comput. Commun. Appl..

[12] Hettich D., Olson M., Jackson A., Kaabouch N. Breast Cancer: Classification of Tumors Using Machine Learning Algorithms. Proceedings of the 2021 IEEE International Conference on Computational Intelligence and Virtual Environments for Measurement Systems and Applications (CIVEMSA).

[13] Beacher F., Mujica-Parodi L.R., Gupta S., Ancora L.A. (2021). Machine Learning Predicts Outcomes of Phase III Clinical Trials for Prostate Cancer. Algorithms.

[14] Vannier M., Butterfield R., Jordan D., Murphy W., Levitt R., Gado M. (1985). Multispectral analysis of magnetic resonance images. Radiology.

[15] Taxt T., Lundervold A. (1994). Multispectral analysis of the brain using magnetic resonance imaging. IEEE Trans. Med. Imaging.

[16] Smistad E., Falch T.L., Bozorgi M., Elster A., Lindseth F. (2015). Medical image segmentation on GPUs—A comprehensive review. Med Image Anal..

[17] Bernal J., Kushibar K., Asfaw D., Valverde S., Oliver A., Martí R., Lladó X. (2019). Deep convolutional neural networks for brain image analysis on magnetic resonance imaging: A review. Artif. Intell. Med..

[18] Mohsen H.M., El-Dahshan E.A., EL-Horbaty E.S.M., Salem A.B.M. (2017). Classification using deep learning neural networks for brain tumors. Future Comput. Inform. J..

[19] Irmak E. (2021). Multi-Classification of Brain Tumor MRI Images Using Deep Convolutional Neural Network with Fully Optimized Framework. Iran. J. Sci. Technol. Trans. Electr. Eng..

[20] Narmatha C., Eljack S.M., Tuka A., Manimurugan S., Mustafa M.Z. (2020). A hybrid fuzzy brain-storm optimization algorithm for the classification of brain tumor MRI images. J. Ambient. Intell. Humaniz. Comput..

[21] Pugalenthi R., Rajakumar M.P., Ramya J., Rajinikanth V. (2019). Evaluation and Classification of the Brain Tumor MRI using Machine Learning Technique. J. Control. Eng. Appl. Inform..

[22] Kang J., Ullah Z., Gwak J. (2021). MRI-Based Brain Tumor Classification Using Ensemble of Deep Features and Machine Learning Classifiers. Sensors.

[23] Khan H.A., Jue W., Mushtaq M., Mushtaq M. (2020). Brain tumor classification in MRI image using convolutional neural network. Math. Biosci. Eng. MBE.

[24] Chelghoum R., Ameur I., HameurLaine A., Jacquir S. (2020). Transfer Learning Using Convolutional Neural Network Architectures for Brain Tumor Classification from MRI Images. Artif. Intell. Appl. Innov..

[25] Stember J.N., Shalu H. (2021). Deep reinforcement learning-based image classification achieves perfect testing set accuracy for MRI brain tumors with a training set of only 30 images. arXiv.

[26] Afshar P., Mohammadi A., Plataniotis K. Brain Tumor Type Classification via Capsule Networks. Proceedings of the 2018 25th IEEE International Conference on Image Processing (ICIP).

[27] Furuta R., Inoue N., Yamasaki T. Fully Convolutional Network with Multi-Step Reinforcement Learning for Image Processing. Proceedings of the AAAI.

[28] Shakeel M., Bajwa N., Anwaar A.M., Sohail A., Khan A., Khan H. Detecting Driver Drowsiness in Real Time Through Deep Learning Based Object Detection. Proceedings of the IWANN.

[29] Gardner D., Akil H., Ascoli G.A., Bowden D.M., Bug W.J., Donohue D.E., Goldberg D.H., Grafstein B., Grethe J.S., Gupta A. (2008). The Neuroscience Information Framework: A Data and Knowledge Environment for Neuroscience. Neuroinformatics.

[30] Menze B.H., Jakab A., Bauer S., Kalpathy-Cramer J., Farahani K., Kirby J.S., Burren Y., Porz N., Slotboom J., Wiest R. (2015). The Multimodal Brain Tumor Image Segmentation Benchmark (BRATS). IEEE Trans. Med. Imaging.

[31] IXI Brain Dataset. https://brain-development.org/ixi-dataset/.

[32] Cheng J. Brain Tumor Dataset. https://figshare.com/articles/dataset/brain_tumor_dataset/1512427.

[33] Kaiser L., Gomez A.N., Chollet F. (2018). Depthwise Separable Convolutions for Neural Machine Translation. arXiv.

[34] Kar A., Biswas P.K. Fast Bayesian Uncertainty Estimation and Reduction of Batch Normalized Single Image Super-Resolution Network. Proceedings of the 2021 IEEE/CVF Conference on Computer Vision and Pattern Recognition (CVPR).

[35] Alshehhi R., Alshehhi A. Quantification of Uncertainty in Brain Tumor Segmentation using Generative Network and Bayesian Active Learning. Proceedings of the VISIGRAPP.

[36] Dosovitskiy A., Fischer P., Ilg E., Häusser P., Hazirbas C., Golkov V., Smagt P.V.D., Cremers D., Brox T. FlowNet: Learning Optical Flow with Convolutional Networks. Proceedings of the 2015 IEEE International Conference on Computer Vision (ICCV).

[37] Carneiro T., Nóbrega R.V.M.D., Nepomuceno T., Bian G., de Albuquerque V.H.C., Filho P. (2018). Performance Analysis of Google Colaboratory as a Tool for Accelerating Deep Learning Applications. IEEE Access.

[38] Pashaei A., Sajedi H., Jazayeri N. Brain Tumor Classification via Convolutional Neural Network and Extreme Learning Machines. Proceedings of the 2018 8th International Conference on Computer and Knowledge Engineering (ICCKE).

[39] Kurup R., Sowmya V., Soman K.P. Effect of Data Pre-processing on Brain Tumor Classification Using Capsulenet. Proceedings of the ICICCT-2019.

